# Supplementary figures and images for: Constitutive gene expression differs in three brain regions important for cognition in neophobic and non-neophobic house sparrows (Passer domesticus)
Source: PLoS One. 2022 May 10;17(5):e0267180. doi: 10.1371/journal.pone.0267180 (PMC9089922; doi:10.1371/journal.pone.0267180)

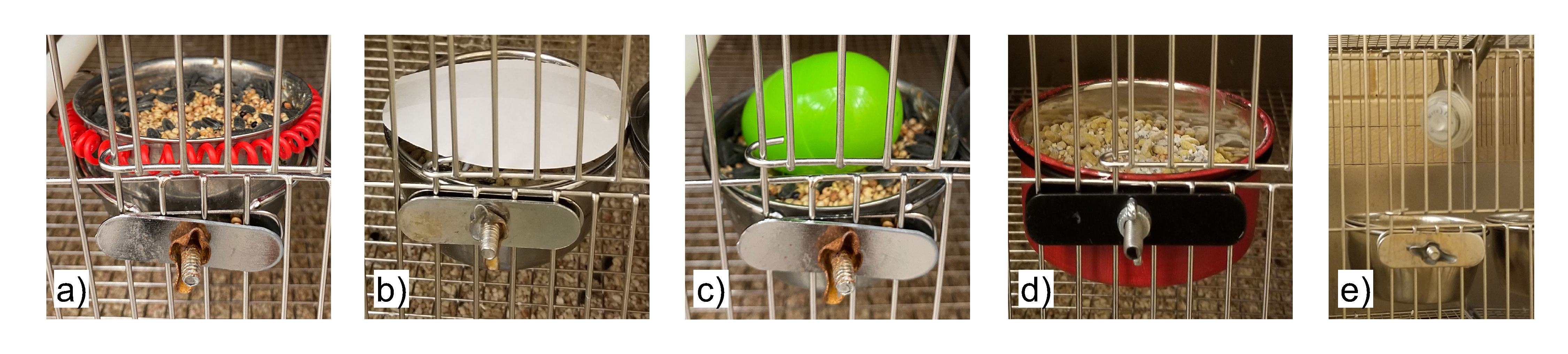

Supplement: S1 Fig — All objects used were placed on, in, or near the normal food dish during 1 h trials. Each house sparrow saw four of the following five objects: a) a red keychain around the edge of the dish, b) a white plastic cover over the dish, c) a green plastic egg in the dish, d) the normal dish painted red, e) a flashing light clipped over the dish. (TIF) [file pone.0267180.s001.tif]

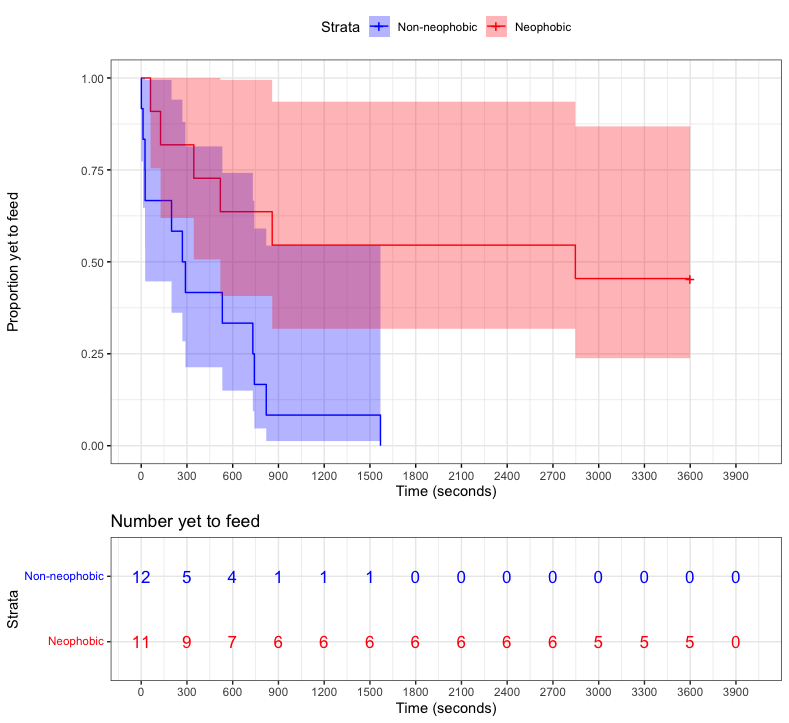

Supplement: S2 Fig — Top: Kaplan-Meier survival curves of house sparrow feeding likelihood in the presence of a novel object for the subset of birds used in RNAseq. There were four object trials for each bird, except for one missing trial from a non-neophobic bird where the video camera malfunctioned. Data are split by neophobia phenotype (non-neophobic n = 3, neophobic n = 3) and with 95% confidence intervals. Bottom: Risk table indicating the number of sparrows yet to feed from the dish in 300 s intervals (trial ended at 3600 s). Both plot and table were created using the ‘survminer’ package in R Studio [71]. (TIF) [file pone.0267180.s002.tif]
