# Supplementary material for: Constitutive gene expression differs in three brain regions important for cognition in neophobic and non-neophobic house sparrows (Passer domesticus)
Source: PLoS One. 2022 May 10;17(5):e0267180. doi: 10.1371/journal.pone.0267180 (PMC9089922; doi:10.1371/journal.pone.0267180)
Supplement: S1 Table — Food dishes were weighed immediately after the lights in the bird room turned off in the evening. Dishes were re-weighed just before the lights turned on in the morning. Each bird was individually housed in a cage with a single food dish. Any differences in food cup mass between night and morning appear attributable to normal variation in mass measurements from the scale used (Mettler Toledo TLE3002E). (DOCX) [file pone.0267180.s003.docx]

| **Sparrow ID** | **Night food cup mass** | **Morning food cup mass** | **Mass difference** |
| --- | --- | --- | --- |
| 87 | 46.65 | 46.65 | 0 |
| 88 | 46.07 | 46.08 | -0.01 |
| 91 | 46.37 | 46.38 | -0.01 |
| 95 | 46.64 | 46.60 | 0.04 |
| 94 | 47.30 | 47.31 | -0.01 |
| 106 | 45.43 | 45.41 | 0.02 |
| 112 | 47.65 | 47.65 | 0.00 |
| 107 | 46.42 | 46.43 | -0.01 |
| 98 | 47.08 | 47.08 | 0 |
| 110 | 46.39 | 46.39 | 0 |
| 116 | 45.24 | 46.23 | -0.99 |
| 144 | 46.96 | 46.96 | 0 |
| *Average:* | *46.52* | *46.59* | *-0.08* |
